# Community-Centered Health Home: Life on the Other Side of the Wall

**DOI:** 10.5888/pcd15.170510

**Published:** 2018-05-24

**Authors:** Sandra Donaldson

Families living in poverty face barriers that threaten their fundamental life-sustaining needs. The crisis-of-the-moment (eg, hunger, shelter) can take immediate precedence over education, environmental conditions, and preventive health care. Maslow’s hierarchy of needs suggests that socioeconomic groups differ in how they prioritize their daily needs, including healthful food choices (1). The socioeconomic conditions in which families live may pose barriers to achieving favorable health outcomes. Social determinants of health are “conditions in the environments in which people are born, live, learn, work, play, worship, and age that affect a wide range of health, functioning, and quality-of-life outcomes and risks” (2). Communities must assess these complex and interconnected determinants of health to identify root causes of health problems. Cross-sector collaborations are necessary for assessing these determinants and building healthier communities to support children and families (3).

Escambia Community Clinics (ECC), a federally qualified health center based in Pensacola, Florida, was one of 5 Gulf Coast community health centers to participate in the Louisiana Public Health Institute’s (LPHI’s) Community-Centered Health Home Demonstration Project (4,5). The community-centered health home model was developed by the Prevention Institute in 2011 as a framework for community health centers to engage in creating safer, healthier, and more equitable communities (6). The model addresses the underlying factors that affect injury and illness, such as the environment, poverty, and safety, to improve health equity and reduce the need for medical treatment (6). ECC adopted the model as a baseline diagnosis approach to target and remedy these underlying factors. The community-centered health home concept recognizes that the goal of the community health center is to tailor care to the sociodemographics of the neighborhood in which the community health center is located (4).

LPHI developed a pilot of this model under the Gulf Region Health Outreach Program, funded by the Deepwater Horizon Medical Benefits Class Action Settlement. With guidance from LPHI, ECC embraced the community-centered health home model and applied it to a local Pensacola school, C.A. Weis Elementary School. ECC and 3 additional core partnering agencies signed a 20-year community school partnership agreement to embed health care, behavior health services, and social service support directly inside the school. A community school partnership offers students and families extended resources, while promoting academics, community, and family engagement.

ECC’s role is to serve the health care needs of students through a pediatric primary care practice within the school. A pediatrician and public health expert defined the scope and vision of the demonstration project, wherein the federally qualified health centers are challenged, as the primary service delivery organizations, to use their position of influence in the community to improve the social, environmental, and economic conditions that determine health (7).

This essay describes how partnering agencies in the Demonstration Project collaborated to work toward solving 2 common problems among families living in poverty: food insecurity and substandard housing. Phase I of the project identified community health indicators, as these community health indicators demonstrated trends related to food insecurity, safety, and housing. Phase II of the project addressed these indicators.

## Food Insecurity

Families living in poverty often have limitations in accessing affordable, healthful food in their communities, whereas fast food restaurants and food with little to no nutritional value from nearby overpriced convenience stores are often readily available. These limitations include safety issues (eg, high levels of crime, unsafe sidewalks, crossing high-traffic intersections by foot with children), access to community resources (eg, lack of direct transportation to and from the grocery store), and lack of information about health-promoting resources (eg, health and wellness programs such as healthy shopping and cooking education) (8). Case studies report that access barriers to stores offering fresh fruits and vegetables are directly linked to high levels of health deprivation (9).

In October 2016, ECC leveraged its partnership in the Weis Community Partnership School by offering supportive services in the community of Oakwood Terrace, a low-income housing community in which many of the school’s students reside. ECC learned from the school’s staff members that 42% of the students attending the Weis Community Partnership School received a backpack of food each Friday, donated by two local churches, to provide additional food throughout the weekend. Recognition of the need for a weekend supply of food sparked concern that some of the school’s families may be experiencing a food shortage. ECC decided to use LPHI supplemental funds to provide more food to these students through a 6-month Food Extension Program.

The 6-month Food Extension Program, developed in partnership with Weis Community Partnership School, Manna Food Pantries, the University of West Florida, and the University of Florida IFAS Extension/Expanded Food and Nutrition Education Program, provided bimonthly food boxes, health education sessions, and cooking classes to families who attended the classes at the Oakwood Terrace Community Center. In addition to bus passes to assist with transportation to grocery stores offering fresh produce and healthful food choices, participants received free cooking tools, such as crock pots and cookbooks, to prepare meals for under $7.00. Manna Food Pantry is a local food distributor that provided food boxes containing basic staples: rice, beans, and fresh fruits and vegetables. The Expanded Food and Nutrition Education Program is a free nutrition education program that teaches participants skills and strategies to stretch food dollars, eat nutritious meals, and improve overall health (10). The class was designed to meet families where they are: it was hosted onsite in the low-income housing project where many of the children attending the Weis Community Partnership School live. Thus, the program connected the school to the housing community, two important hubs in the community.

## Substandard Housing

Oakwood Terrace is a privately owned US Department of Housing and Urban Development Section 8 apartment complex that comprises 300 apartments. Approximately 200 of the more than 500 students attending Weis Community Partnership School live in this complex. Surrounded by an 8-foot cement wall, Oakwood Terrace is in one of Escambia County’s most socially vulnerable zip codes. *Life on the other side of the wall* is nothing short of surviving severe socioeconomic deprivation. In 2015, 11% of households in Escambia County were living in poverty as determined by the yearly federal poverty guidelines. In addition, 27% of children in Escambia County, compared with 23% of children in Florida, were living in poverty (11). Poor housing and poor health create a need for a targeted public investment. Intervention studies link housing improvements to health improvements, and housing improvements may promote improved social relationships within and beyond the household (12).

ECC, in collaboration with the University of West Florida, conducted a survey among residents of Oakwood Terrace to identify social problems in the complex. The survey addressed topics such as housing conditions and access to healthful foods. Eighteen women and 2 men representing more than 70 family members aged 0 to 71 participated in the survey. The survey results showed that housing conditions were substandard and the complex was riddled with crime. Poor insulation of the apartment units led to high utility bills, resulting in utilities being disconnected. Food could not be stored in apartments that did not have utilities, and families that did not have utilities could have faced eviction from their apartment. Families found themselves living within this vicious cycle of crisis.

After examining the survey results, ECC shared the survey information with the owners of the complex, who live out of state, and the two groups established a working relationship. This relationship bridged the gap between residents and management. The owners learned about the needs of their residents and began to implement the changes that were most important to them. In addition, the owners hired a new on-site property management team. This event set the stage for new collaborative momentum to implement a spectrum of components for transforming the community.

## Future Directions

The philosophy of the community-centered health home is that the health care institution has a voice that is well-regarded and that can affect local policy or community conditions. The actions taken by community-centered health homes typically include partnering with external organizations and a diverse group of community stakeholders through a two-way relationship (13). As such, fostering partnerships and enhancing community engagement were the driving forces behind ECC’s role throughout the Demonstration Project.

The Demonstration Project continued to address the food insecurity crisis and also looked more closely at the housing survey responses to determine further underlying adverse conditions. The owners integrated funding resources with ECC to sustain and expand the food extension program. Apartment renovations began in 2016 and are scheduled through 2018. Most recently, apartment windows were tinted to reduce apartment temperatures during the warm months, lower utility bills, and improve aesthetics. Hands-on education for residents on cleaning and sanitizing units will help maintain newly updated apartments and assist in instilling a sense of ownership and pride among residents. Health screenings were offered, along with continued nutritional education, adult educational resources, employment enrichment, public transportation education, and plans for a resident-managed community garden.

As part of Phase II, we conducted a second survey. The survey was conducted among participants attending the Food Extension Program in June 2017. The purpose of the survey was to identify the collective impact and outcomes of the program. Fifteen residents participated in the survey. These survey results showed that families had increased their monthly vegetable intake by more than half since the first survey was administered. However, intake of items such as cakes, cookies, and sugar-sweetened beverages decreased only slightly; these items are still readily available to families at the local corner convenience store, the only store in the neighborhood that offers immediate access to any kind of food. This survey also showed that transportation continued to affect the ability to make healthful decisions about food. A transportation option, now in the planning stages, will offer paratransit routes directly to and from Oakwood Terrace to the grocery store, with the transportation expense paid by a private donor.

The owners invested more than $1 million into the safety and renovations of Oakwood Terrace. The newly renovated apartments were designed to enhance a sense of community and create physical activity resources to ameliorate a host of health conditions, from obesity to depression. A new playground for children to safely play within the walls of the complex was built as well as a new community center. Before transformation of the complex, families kept their children in poorly insulated apartments in fear of high levels of crime. New security measures to improve safety at Oakwood Terrace include a 7-day-per-week police presence and an internet surveillance system. Additionally, some families are now inspired to apply what was learned throughout the program by migrating from Oakwood Terrace’s low-income housing to affordable housing and eventually homeownership.

In June 2017, Morgan Cox, partner of DM Oakwood Terrace, LLC, owners of Oakwood Terrace, stated, “We are so thrilled about the progress taking place at Oakwood Terrace, and it is a testimony to public and private entities joining forces and working together.”

Systemic cultural and social norms take time to transform into an acceptance of change. The concrete wall surrounding the community of Oakwood Terrace continues to stand, but there is now a beacon of hope shining through as that wall begins to be disassembled one metaphorical brick at a time.
